# Impact of Cabin Ozone Concentrations on Passenger Reported Symptoms in Commercial Aircraft

**DOI:** 10.1371/journal.pone.0128454

**Published:** 2015-05-26

**Authors:** Gabriel Bekö, Joseph G. Allen, Charles J. Weschler, Jose Vallarino, John D. Spengler

**Affiliations:** 1 International Centre for Indoor Environment and Energy, Department of Civil Engineering, Technical University of Denmark, Lyngby, Denmark; 2 Department of Environmental Health, Harvard School of Public Health, Boston, Massachusetts, United States of America; 3 Environmental and Occupational Health Sciences Institute, Rutgers University, Piscataway, New Jersey, United States of America; The Ohio State University, UNITED STATES

## Abstract

Due to elevated ozone concentrations at high altitudes, the adverse effect of ozone on air quality, human perception and health may be more pronounced in aircraft cabins. The association between ozone and passenger-reported symptoms has not been investigated under real conditions since smoking was banned on aircraft and ozone converters became more common. Indoor environmental parameters were measured at cruising altitude on 83 US domestic and international flights. Passengers completed a questionnaire about symptoms and satisfaction with the indoor air quality. Average ozone concentrations were relatively low (median: 9.5 ppb). On thirteen flights (16%) ozone levels exceeded 60 ppb, while the highest peak level reached 256 ppb for a single flight. The most commonly reported symptoms were dry mouth or lips (26%), dry eyes (22.1%) and nasal stuffiness (18.9%). 46% of passengers reported at least one symptom related to the eyes or mouth. A third of the passengers reported at least one upper respiratory symptom. Using multivariate logistic (individual symptoms) and linear (aggregated continuous symptom variables) regression, ozone was consistently associated with symptoms related to the eyes and certain upper respiratory endpoints. A concentration-response relationship was observed for nasal stuffiness and eye and upper respiratory symptom indicators. *Average* ozone levels, as opposed to peak concentrations, exhibited slightly weaker associations. Medium and long duration flights were significantly associated with more symptoms compared to short flights. The relationship between ultrafine particles and ozone on flights without meal service was indicative of ozone-initiated chemistry.

## Introduction

Ozone is a well-recognized respiratory irritant [1]. The health effects include various mortality and morbidity endpoints, including respiratory symptoms, hospital admissions for respiratory diseases and the exacerbation of asthma [2–10]. Additionally, adverse effects have been linked not only to exposure to ozone itself, but also to the byproducts of ozone-initiated chemistry [11]. Products of these reactions can impact perceptions of indoor air quality and may lead to health effects (e.g. eye irritation, irritation of the upper airways, airflow limitation in the conducting airways) [12–16]. Many of the studies documenting the impact of ozone and its products on health symptoms have been conducted in the laboratory. A field study targeting 100 US office buildings by Apte et al. [17] confirmed some of the earlier laboratory findings. The authors reported positive relationships between late workday outdoor ozone concentrations (mean ~33 ppb, range 2–98 ppb) and upper respiratory symptoms, dry eyes, headache and neurological symptoms (fatigue or trouble concentrating). 24-h average ozone concentrations and average workday concentrations were significantly associated with having at least one upper respiratory symptom. The positive correlation between outdoor ozone levels and the concentration of aldehydes within the building indicated the occurrence of ozone chemistry.

Commercial airplanes routinely cruise in the upper troposphere or the lower stratosphere, where ozone can reach concentrations of hundreds of parts per billion [18, 19]. Ozone entering the airplane cabin can lead to elevated concentrations (levels exceeding 100 ppb, peak levels exceeding 200 ppb) and increased exposure for passengers and crew [20–23]. Ozone can be removed from the cabin by catalytic converters. However, many aircraft do not use ozone converters. The converters in use do not always perform well due to surface “poisoning” by various contaminants or imperfect re-furbishing of catalysts during scheduled replacement. Current regulation allows 250 ppb peak ozone concentrations and 100 ppb 3-hour ozone levels in aircraft cabins [24]. These limits exceed the US EPA National Ambient Air Quality Standard (NAAQS) for ground level ozone of 75 ppb over an 8 h averaging time and the EPA Clean Air Science Advisory Committee recommendation for the NAAQS human-health based standard of between 60 and 70 ppb. Effects of ozone and its reaction products on occupants may occur at even lower levels [13, 17, 25, 26].

The most recent studies of aircraft environments have investigated the health consequences of long term exposure of flight attendants to tobacco smoke during the years when smoking was allowed on flights, exposure to semi-volatile chemicals in the cabin, and self-reported health of flight attendants in comparison to the general population [27–30]. Earlier studies have focused on general indoor air quality in aircraft cabins, especially on low humidity, and reported health problems. Most of these studies were performed before smoking was banned and their focus was on crew, especially flight attendants [31–34]. Several studies also focused on ozone in airplane cabins. Ozone-related symptoms were significantly more frequent on aircraft that flew at high altitudes, presumably due to higher ozone concentrations [35, 36]. Ozone’s potential effects on the human visual system responses in aircraft were indicated by Daubs [37]. Passengers in a simulated aircraft cabin reported less satisfaction with the air quality and more symptoms during conditions with elevated (60–70 ppb) ozone concentrations [26].

There is a need to better understand ozone exposures on actual commercial aircraft operating across the planet under today’s flight conditions and the impact ozone has on passengers’ health and comfort. The aim of this study was to i) monitor the indoor environmental parameters, including ozone concentration, on 83 US domestic and international flights within and between Asia, Australia and the Americas, ii) monitor the passengers’ assessment of the indoor environment and their own symptoms on these flights, and iii) evaluate the dataset for associations between cabin environmental parameters and passenger health and comfort.

## Materials and Methods

### Measurements

The cabin air of 83 flights was monitored between February 2008 and August 2010 as a component of a study of onboard environmental conditions and passenger and crew responses. These transcontinental and transoceanic flights were operated by three different airlines. Ozone concentrations were measured with a 2B Tech model 205 ozone monitor (2B Technologies Inc., Boulder, Colorado, USA). Carbon dioxide concentrations were measured using a 7565 Qtrak monitor (TSI Inc., Shoreview, MN, USA.). The particle number concentrations (PNC) in the size range between 6 nm and 3 μm, representing mainly ultrafine particles (UFP), were measured with a water-based condensation particle counter Model 3781 (58 flights; TSI Inc., Shoreview, MN, USA). The other environmental parameters included relative humidity (RH), cabin pressure and temperature (T) (7565 Qtrak monitor, TSI Inc.). All instruments used onboard were electro-magnetic interference (EMI) certified at Boeing (Everett, WA). The sensors were also tested for performance under pressure flight conditions at Battelle (Columbus, OH). The measurements were conducted by a project engineer onboard the aircraft. Monitors were located either in an aisle seat or a middle seat in the middle of economy class. Instruments with pumps and batteries were positioned under the seat. Measurements were recorded continuously, at one-minute intervals, from 10,000 feet ascent through 10,000 feet descent. Towards the end of the flight, passengers on 80 flights were asked to complete a questionnaire with focus on a wide range of symptoms and satisfaction with the indoor climate. The questionnaire was developed by a team of medical and public health experts. It was designed to capture a broad range of potential adverse human health effects plausibly related to the environmental parameters measured (e.g., particles, ozone, volatile organic compounds, ventilation) based on the extensive literature on these factors in buildings and their impact on building occupants.

Information on flight duration, aircraft model, aircraft capacity and occupancy loads was collected. Latitude categorization (1.polar, 2.equatorial) was based on the flight path. Each flight route was classified by the fraction of flight time north of 35°N. Flights that followed northern routes (polar, higher latitudes) were those that had fractions higher than 0.5. Flight duration was categorized into: 1.short (shorter than 3 hours), 2. medium (between 3 to 6 hours) and 3. long (longer than 6 hours). The aircraft type was treated as a categorical variable with categories 1. 737, 2. 747, 3. 767, 4. 777 (all Boeing) and 5. Airbus. Due to the small number of Airbuses (two A-340 and four A-380), these were pooled into one category.

### Ethics Statement

This study was reviewed and approved by the Institutional Review Board (IRB) at Harvard University and Battelle Memorial Institute, a collaborating organization for the overall project (Protocol #FN006904-FTSUR and #FN006904-CSURV). Harvard School of Public Health had an IRB Authorization Agreement with the IRB of Battelle Memorial Institute (Protocol #P16992 and #P18875). The Battelle IRB gave a waiver of the written informed consent because the only recording of the participant’s identity would have been the consent form. The consent statement was read over the public address system of the plane. People who did not give consent expressed that by not completing a survey. Positive consent was completing the survey.

### Data analyses

PNC and corresponding ozone levels were analyzed for all available data and for three different periods on each flight, established based on the distribution of the measured data: while the 10-minute running mean PNC i) exceeded 5000 particles per cm^3^ (cm^-3^ throughout the paper); ii) was between 500 and 5000 cm^-3^, and iii) was below 500 cm^-3^. A UFP “event” corresponded to the interval between a sharp excursion in the indoor PNC until its return, usually rapid, to the original level. Major events were those during which the PNC exceeded 5000 cm^-3^, minor events were those during which the PNC reached a concentration between 500 and 5000 cm^-3^. Mean PNC and corresponding ozone concentrations were calculated for each UFP event. The relationship between UFP and ozone was analyzed separately for flights with and without meal service.

Outside ventilation rate per person (Q_pers_) was calculated from the measured CO_2_ concentrations using the constant concentration method [38]. This method requires the CO_2_ to reach steady state. CO_2_ levels were relatively stable in–flight. In the calculations we used an outdoor CO_2_ concentration of 386 ppm, which is the mean global outdoor CO_2_ concentration for the six months in which onboard sampling was performed, as reported by National Oceanic and Atmospheric Administration. This value reasonably represents the outdoor CO_2_ concentrations at flight altitudes [39]. We used 18 L/h for the CO_2_ generation rate per person [40].

The analyses of the associations between ozone and symptoms were performed on the population of all passengers (n = 4174), both on individual and grouped symptoms. The individual symptoms included: watery eyes; itchy eyes; dry eyes; blurred, dim, altered vision; eye pain; nasal stuffiness; runny nose or sneezing; dry irritated or sore throat; hoarseness/loss of voice; cough; dry mouth or lips; headache; lightheaded, dizzy or faint. The following aggregate symptom categories were constructed for analyses:

- Eye, mouth symptoms: watery eyes; itchy eyes; dry eyes; blurred, dim, altered vision; eye pain; dry mouth or lips
- Upper respiratory: nasal stuffiness; runny nose or sneezing; sinus pain, pressure, congestion; dry, irritated or sore throat; hoarseness/loss of voice; cough; loss of smell or taste; nose bleed
- Ear, head symptoms: ringing in the ears; decreased hearing; pain, pressure or blockage in the ears; headache; lightheaded, dizzy or faint
- Muscular symptoms: back pain or stiffness; swelling in the lower extremities; pain in legs or feet; calf pain; tingling in lower extremities; unusual tiredness or fatigue; loss of coordination or balance; stiff or painful neck; tingling in face or lips; shoulder pain or stiffness; arm pain or stiffness; hand pain or stiffness; numbness or tingling in hands; shaking or trembling in hands; muscle weakness, achiness
- Lower respiratory symptoms: shortness of breath or difficulty breathing; chest tightness or pressure; chest pain
- Digestive symptoms: indigestion; bloating/gas/cramps/pressure; diarrhea; constipation; nausea/vomiting; stomach ache; urinary frequency
- Neurological symptoms: difficulty concentrating; confusion, difficulty finding words/thinking/counting; apathy, loss of motivation, depression; anxiousness, irritability; sleep disturbances—inability to stay awake/go to sleep

Aggregate categories were defined as the presence of at least one of the respective symptoms in the group (dichotomous variables “Any…symptom”). Using slightly altered symptom grouping, we also calculated the number of symptoms reported by each passenger (continuous variable), as well as rating of the quality of indoor air and satisfaction with odor and air freshness.

Separate analyses of the associations between ozone and passenger reported symptoms were performed on overall symptom descriptors calculated for each flight (continuous variables, n = 80). These included the prevalence of the individual symptoms, average and maximum number of symptoms reported per person on each flight, average number of symptoms within the above mentioned groups, the prevalence of any symptom in these groups (number of passengers reporting at least one symptom within the category, divided by the number of passengers who filled in the questionnaire on the flight) and average rating of the quality of indoor air and average satisfaction with odor and air freshness.

### Statistical analyses

For statistical analyses STATA 11.2 for Windows was used (StatCorp LP, College Station, TX, USA). Spearman correlation coefficients were calculated for the measured environmental parameters, as some of them were not normally distributed (Shapiro-Wilk’s test for normality). Differences in ozone concentrations between different flight characteristics were tested with non-parametric two-sample Wilcoxon rank-sum test. The relationship between ozone concentrations and symptoms were analyzed using adjusted (multivariate) regression models. Logistic regression was used for dichotomous dependent variables. On continuous dependent variables, linear regression was used. In the initial analyses, all of the following covariates were used in the models (full models): average temperature, relative humidity, cabin pressure, ventilation rate, percent occupancy, airline, flight duration, aircraft type, sex, smoking habits (regular, social, none) and age (categorical: less than 20; 20–29; 30–39; 40–49; 50–59; 60–69; 70 or over). Statistical models looking for associations between ozone and the overall symptom descriptors calculated for each flight (n = 80) did not include smoking habits, sex and age. These analyses were completed both on non-transformed ozone concentrations (scaled by a factor of 10 to make interpretation of results easier) and on Ln-transformed data, since ozone concentrations followed a log-normal distribution. Additionally, a categorical variable for ozone with five levels of exposure (quintiles) was used to assess potential concentration–response relationships between ozone and reported symptoms. To determine the overall significance of the linear concentration-response relationship, the quintile categories (1–5) were treated as a continuous variable.

In a separate set of analyses, stepwise backward regression was applied to identify significant predictor variables with inclusion criteria of p<0.2 [41]. The associations between ozone and symptoms were then adjusted only for the selected descriptors. These analyses were completed on Ln-transformed data. Both average and peak ozone concentrations were tested in separate analyses. In all statistical analyses a value of p<0.05 was considered statistically significant.

## Results and Discussion

### Environmental parameters

Table 1 shows the results of the measurements performed on the 83 flights, while Table 2 stratifies the environmental parameters on airline, aircraft type, flight duration, latitude and occupancy. Temperature and relative humidity were relatively similar across the flights. The average humidity was low, as reported in several other studies [31, 42, 43]. Lower RH was measured on long flights compared to shorter flights and on flights with less than 75% occupant load compared to those with higher occupancy. The FAA Federal Aviation Regulation (FAR) 14CFR25.841 [44] states that the minimum cabin pressure under normal operating conditions should not be less than the pressure found at an altitude of 8,000 feet (75.3 kPa). The mean cabin pressure across all flights was 80.8 kPa, and the highest “average per flight” was 89 kPa. None of the 83 flights had pressures below 75.3 kPa. The average CO_2_ concentration exceeded the recommended level of 1000pm on 89% of the flights, comparable to the 87% reported by Nagda et al. [45]. Lower CO_2_ levels were previously reported by Hagighat et al. [42], Lee et al. [43], Lindgren and Norbäck [31], especially during cruising conditions compared to non-cruising conditions.

**Table 1:** Descriptive statistics of the measured parameters (based on averages per flight) and occupancy.

	n	Mean	SD	Median	Min	Max
**Mean T (°C)**	83	24.4	1.8	24.6	19.2	31.3
**Mean RH (%)**	83	12.2	5.3	11.3	2.7	36.2
**Mean Pressure (kPa)**	83	80.8	2.5	80.4	76.4	88.9
**Mean CO**_**2**_ **(ppm)**	83	1373	303	1361	573	2039
**Mean Ozone (ppb)**	83	16.1	17.3	9.5	0.01	110.4
**Max Ozone (ppb)**	83	38.9	39.2	29.1	0.3	256
**Q**_**pers**_ **(L/s/person)**	83	5.8	3.1	5.1	3.0	28.0
**Mean PNC (cm** ^**-3**^ **)**	58	982	3981	64.0	0.1	22052
**Occupancy (%)**	81	75.5	23.1	82.0	23.0	100

**Table 2:** Flight characteristics and corresponding environmental parameters (based on averages for each flight).

Variable	n(%)	Mean T (°C)	Mean RH (%)	Mean Pressure (kPa)	Mean CO_2_ (ppm)	Median O_3_ (ppb)
**Airline**						
**1**	17(20.5)	23.8	14.7	81.2	1469	9.5
**2**	42(50.6)	24.9	11.1	79.7	1386	11.6
**3**	24(28.9)	24.0	12.3	82.5	1284	9.4
**Aircraft type (mean capacity)**						
**B737 (138)**	44(53.0)	24.9	11.4	79.7	1409	10.3
**B747 (325)**	10(12.0)	24.3	10.0	83.7	1149	9.7
**B767 (239)**	10(12.0)	23.8	14.3	82.5	1262	14.2
**B777 (305)**	13(15.7)	24.2	13.8	81.1	1542	11.2
**Airbus (360)**	6(7.2)	22.4	14.9	81.0	1307	1.6
**Duration**						
**Short**	33(39.8)	24.1	14.5	80.9	1425	13.6
**Medium**	35(42.2)	25.0	11.0	79.8	1394	9.5
**Long**	15(18.1)	23.9	10.0	82.8	1212	9.3
**Latitude**						
**Polar**	35(42.2)	24.9	10.3	80.3	1340	16.7
**Equatorial**	48(57.8)	24.1	13.6	81.2	1397	7.5
**Occupancy**						
**≤75%**	34(42.0)	24.2	9.4	80.0	1145	11.9
**>75%**	47(58.0)	24.5	14.2	81.4	1542	9.5

The average ozone concentration across all flights was 16.1 ppb. The highest average ozone concentration within a single flight was 110.4 ppb. Across all flights the mean of the highest value per flight was 38.9 ppb, with the highest peak level reaching 256 ppb for a single flight. The average ozone concentration was above 60 ppb on two flights. Ozone levels on thirteen flights (16%) exceeded 60 ppb while on 10 flights (12%) it exceeded 75 ppb. Consistent with Weisel et al. [46], these concentrations are lower than those reported by Spengler et al. [23] and by studies summarized by the authors. Airplanes encounter higher external ozone levels during the winter and spring and on routes that fly at higher latitudes [18, 19, 22, 23, 47, 48]. Among our flights, 16 were flown in summer months and 67 in winter months. However, the majority of our flights were equatorial. Significantly higher ozone levels were measured on flights that followed higher latitude routes (polar; >35°N) compared to flights that followed lower latitudes (equatorial) (p<0.05). Although without statistical significance, ozone concentrations were lower on medium and long flights compared to short flights, presumably due to the use of ozone convertors on larger planes [23, 46].

Significant correlations were found between occupancy load and relative humidity (ρ = 0.62) and between occupancy load and CO_2_ (ρ = 0.68; S1 Table), as also indicated by Lindgren and Norbäck [31] and Nagda et al. [45]. The strong relationship between CO_2_ concentration and humidity (ρ = 0.60) is consistent with the recent work by Giaconia et al. [49], who highlighted the relationship between relative humidity and carbon dioxide concentration in order to provide acceptable levels of both to the crew and passengers with proper ventilation control and without additional humidification. The strong negative correlation between ventilation rate and occupancy reflects the fact that the ventilation rate was estimated from the occupant-generated CO_2_ concentration.

### Passenger surveys

On average, 52 persons per flight (range 8–97) filled in the questionnaire. Forty six percent of passengers reported at least one symptom related to the eyes or dry mouth. The most common symptoms were dry mouth or lips (26% of passengers), dry eyes (22.1%) and nasal stuffiness (18.9%). Between 5 and 10% of passengers reported having itchy eyes; headache; dry, irritated, sore throat and runny nose or sneezing. A third of the passengers reported at least one muscular symptom or fatigue and the same fraction at least one upper respiratory symptom. One fourth reported a symptom related to the ears, headache or dizziness (Table 3). On average 2.4 symptoms per passenger were reported, most of them being eye/upper respiratory symptoms followed by muscular pain (S2 Table). Lower respiratory symptoms were relatively rare. The average prevalence of symptoms calculated individually for each flight (n = 80) follow similar trends (S3 Table).

**Table 3:** Prevalence of symptoms (%) among passengers (n = 4158).

Symptom	Prevalence
**Dry mouth or lips**	26.0
**Dry eyes**	22.1
**Nasal stuffiness**	18.9
**Itchy eyes**	10.7
**Dry, irritated, or sore throat**	8.3
**Runny nose or sneezing**	8.1
**Headaches**	6.4
**Cough**	6.0
**Watery eyes**	5.6
**Blurred, dim, altered vision**	2.0
**Hoarseness/loss of voice**	1.5
**Lightheaded, dizzy or faint**	1.4
**Eye pain**	1.2
**Any eye, mouth symptom**	46.4
**Any muscular symptom**	34.6
**Any upper resp. symptom**	33.1
**Any ear, head symptom**	24.5
**Any digestive symptom**	9.5
**Any neurol. symptom**	7.3
**Any lower resp. symptom**	1.5

Recent studies of the aircraft environment have focused on the health effects of long-term exposure to cabin environment and secondhand tobacco smoke during the time smoking was allowed on airplanes [27, 50]. Earlier questionnaire studies on symptoms reported while in-flight on commercial aircrafts were conducted before smoking was banned on most commercial flights, and they mainly focused on crew members. Common problems reported among crew were dry skin, nasal symptoms, eye irritation, fatigue, cold, but also sleep disturbances, digestive disturbances and musculoskeletal symptoms, especially on longer flights [32, 33, 51]. Rankin et al. [52] conducted a survey among 3630 passengers on 71 flights. The two health symptoms experienced most by passengers were back/joint/muscle pain and dry or stuffy nose. Thus, earlier studies have consistently reported eye and nasal symptoms along with muscle ache as the most frequent reasons for complaints during flight. Our results are in line with those findings.

### Regression analyses

**Fig 1:**
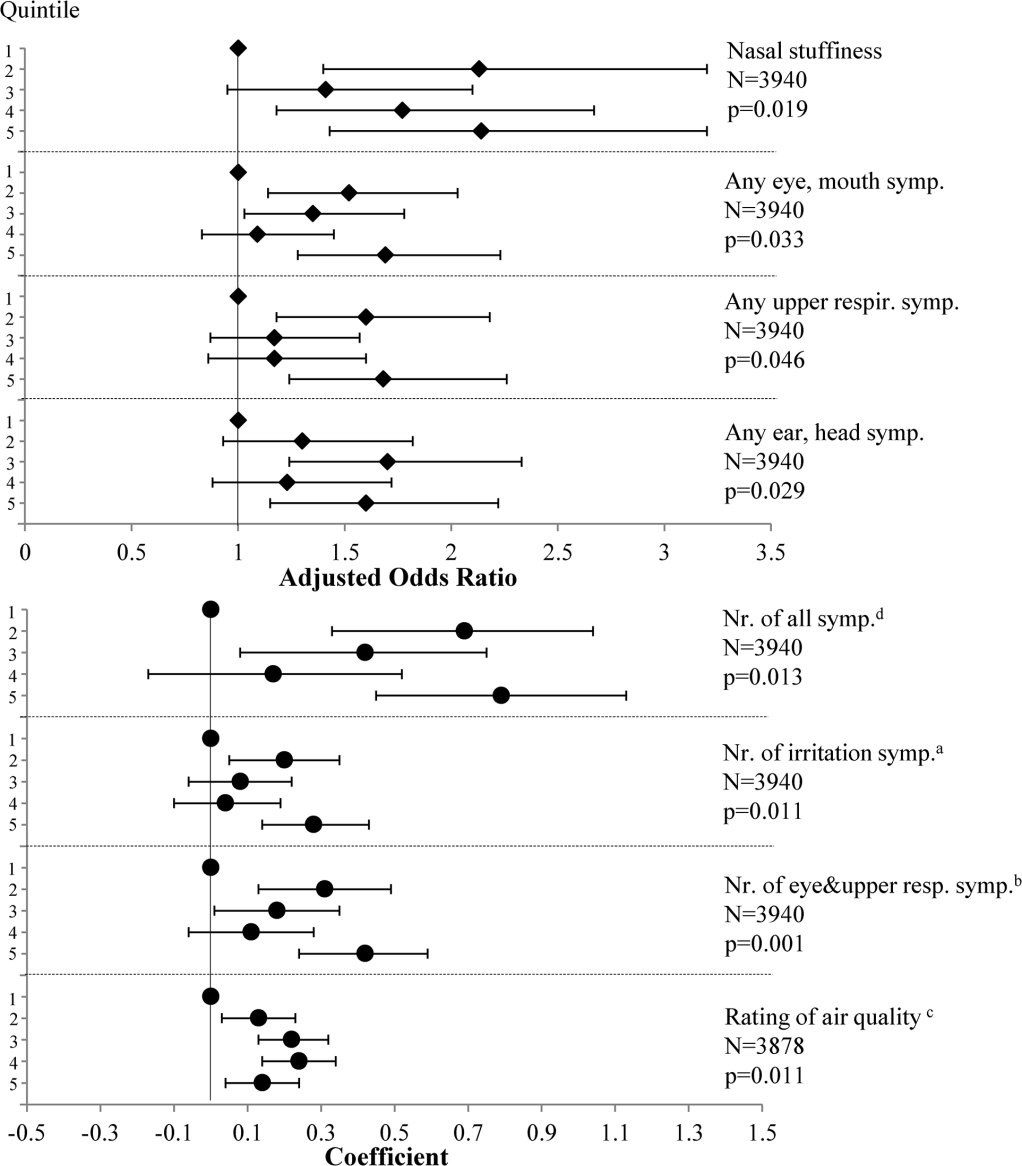
Significant concentration-response relationships between maximum ozone concentrations and reported symptoms/perceptions. Results from logistic regression (aOR, 95% CI) are shown on top, linear regression (coefficient, 95% CI) on the bottom. ^a^ number of the following symptoms: watery eyes, itchy eyes, dry eyes, blurred dim altered vision, eye pain, runny nose or sneezing, dry irritated or sore throat, hoarseness/loss of voice, cough; ^b^ nose bleed and sinus pain/pressure/congestion were included among the ear, head symptoms in these tests, not among eye and upper respiratory symptoms; ^c^ 1 = Very good, 2 = Good, 3 = Adequate, 4 = Poor, 5 = Very poor; ^d^ number of all symptoms in the questionnaire (see full list under various symptom groups in the Methods).

In the full models, ozone concentrations were significantly associated with reports of having a dry mouth or lips, itchy eyes and at least one symptom related to the eyes or mouth. Among the continuous symptom variables, the number of selected irritation symptoms (see superscript “a” in the caption of Fig 1), the number of eye, mouth and upper respiratory symptoms and the rating of air quality were associated with the measured ozone levels (S4 Table). Significant trends in the concentration-response relationship were observed for *maximum* ozone levels (quintiles) and several symptom indicators (Fig 1), especially those related to the eyes, mouth, upper respiratory symptoms, total number of these symptoms, and the number of selected irritation symptoms, which were also driven by eye and upper respiratory symptoms. Both the maximum and the average ozone concentrations exhibited a significant positive concentration-response relationship with the mean rating of the air quality. No other endpoint was associated with *average* ozone levels in a concentration-dependent manner. This may reflect the relatively low average ozone concentrations in the present study, due to the high percentage of flights at equatorial latitudes.

Models using only the significant variables from a stepwise selection procedure indicated similar associations between ozone and symptoms (Tables 4 and 5). Furthermore, satisfaction with odor was lower with increasing peak ozone concentration. Ozone-initiated chemistry in the indoor environment can increase unpleasant odors indoors and decrease the perceived air quality [12, 13]. Similar analyses using ln-transformed *average* ozone concentrations showed significant associations with itchy eyes, having at least one eye/mouth symptom, the number of eye and upper respiratory symptoms and the rating of the air quality. These associations were slightly weaker than the corresponding associations for the peak ozone concentrations (except for itchy eyes). Using peak ozone concentrations can be sensitive to short-duration extreme readings. We therefore tested all relationships presented in S4 Table, Tables 4 and 5 (models where ozone had an effect on symptoms) and in Fig 1 using the 95^th^ percentile of the measured ozone concentrations and the highest 10-minute running average ozone concentration. The results were nearly identical to the analyses based on peak ozone levels. However, the significant concentration-response relationship in Fig 1 was only retained for the “number of eye and upper respiratory symptoms”.

**Table 4:** Associations between Ln-transformed maximum ozone concentration and reported symptoms by stepwise logistic regression.

Dependent variable	N	aOR^a^ (ozone)	95% CI (ozone)	p-value (ozone)	Flight related variables in the final model^b^
**Dry mouth or lips**	4018	**1.14**	**1.05–1.24**	**0.002**	RH, occupancy, airline(3.OR>1), duration
**Dry eyes**	3940	1.08	0.96–1.21	0.20	RH(OR>1), Q_pers_, occupancy(OR<1), airline(2,3.OR>1), duration(2.OR>1), aircraft(2,3,4,5.OR>1)
**Nasal stuffiness**	3981	1.13	1.00–1.28	0.055	T(OR<1), pressure, Q_pers_(OR<1), occupancy(OR<1), airline(2,3.OR>1), duration(2.OR>1), aircraft
**Itchy eyes**	4053	**1.16**	**1.04–1.29**	**0.008**	-
**Dry, irritated or sore throat**	3973	NS	-	-	duration(3.OR>1)
**Runny nose or sneezing**	3971	NS	-	-	T(OR<1), duration(3.OR>0)
**Headaches**	4053	NS	-	-	pressure, duration, aircraft
**Cough**	4078	NS	-	-	airline
**Watery eyes**	4158	NS	-	-	T(OR<1), airline(3.OR>0)
**Blurred, dim, altered vision**	3995	NS	-	-	RH(OR>0), Q_pers_(OR>1), airline
**Hoarseness/loss of voice**	3964	1.29	0.96–1.72	0.09	occupancy
**Lightheaded, dizzy or faint**	3973	1.24	0.92–1.69	0.16	RH(OR>1), pressure, Q_pers_
**Eye pain**	3998	NS	-	-	T, RH(OR<1), aircraft(4,5.OR>1)
**Any eye, mouth symptom**	3940	**1.16**	**1.07–1.26**	**0.000**	T(OR<1), RH(OR>1), pressure(OR>1), Q_pers_(OR<1), occupancy(OR<1), airline(3.OR>1), duration(2,3.OR>1)
**Any muscular symptom**	3981	NS	-	-	pressure, Q_pers_(OR<1), occupancy(OR<1), airline(2.OR>1), duration(2,3.OR>1), aircraft(2.OR>1)
**Any upper resp. symptom**	3940	1.03	0.93–1.14	0.55	T(OR<1), pressure, Q_pers_, occupancy(OR<1), airline(2,3.OR>1), duration(2.OR>1), aircraft
**Any ear, head symptom**	3971	NS	-	-	T(OR<1), RH, aircraft(2,3,4,5.OR<1)
**Any digestive symptom**	4060	NS	-	-	T, pressure, occupancy(OR<1), airline, duration(3.OR>1)
**Any neurol. symptom**	4041	0.94	0.79–1.11	0.46	pressure, Q_pers_, airline(3.OR>1), aircraft(4.OR>1)
**Any lower resp. symptom**	4158	NS	-	-	RH

For all models R^2^<0.1, p<0.05, except Itchy eyes, p = 0.06; Cough, p = 0.12; Watery eyes, p = 0.09; Hoarseness/loss of voice, p = 0.14; Any lower resp. symptom, p = 0.18

^a^ NS—not selected by the stepwise backward variable selection

^b^ The flight-related variables that were included in the final model based on a significance level of p<0.2 for removal from the model (stepwise backwards variable selection [41]). In parentheses the directions of the ORs for the variables (continuous) or their categories (categorical) are indicated when significant at p<0.05.

**Table 5:** Associations between Ln-transformed maximum ozone concentrations and reported number of symptoms in various categories by stepwise linear regression analyses.

Dependent variable	N	Coef.^f^ (ozone)	95% CI (ozone)	p-value (ozone)	Flight related variables in the final model^g^
**Nr. of all symp.** ^**d**^	3940	0.078	-0.04–0.20	0.192	T(coef<0), RH(coef>0), pressure, Q_pers_, occupancy(coef<0), airline(2,3.coef>0), duration(2,3.coef>0), aircraft(4.coef>0)
**Nr. of irritation symp.** ^**a**^	4018	**0.084**	**0.042–0.13**	**0.000**	T, RH(coef>0), Q_pers_, occupancy(coef<0), airline(3.coef>0), duration(2,3.coef>0)
**Nr. of eye&upper resp. symp.** ^**b**^	4018	**0.102**	**0.055–0.15**	**0.000**	T(coef<0), RH, occupancy(coef<0), airline(3.coef>0), duration(2,3.coef>0)
**Nr. of muscular symp.**	4018	NS	-	-	pressure, Q_pers_(coef<0), occupancy, airline(2,3.coef>0), duration(2,3.coef>0), aircraft(2.coef>0)
**Nr. of ear, head symp.** ^**b**^	3971	NS	-	-	T(coef<0), aircraft(2,3,4,5.coef<0)
**Nr. of digestive symp.**	4053	0.009	-0.004–0.022	0.173	T, airline, duration(3.coef>0)
**Nr. of neurol. symp.**	4012	0.007	-0.008–0.023	0.36	pressure, Q_pers_, airline(3.coef>0), aircraft
**Nr. of lower resp. symp.**	4158	NS	-	-	RH, aircraft
**Rating of air quality** ^**c**^	3951	**0.047**	**0.016–0.078**	**0.003**	T(coef>0), pressure(coef<0), occupancy(coef>0), aircraft(2,4.coef<0)
**Satisfaction with odor** ^**e**^	4019	**0.042**	**0.007–0.076**	**0.018**	T(coef>0), RH(coef>0), pressure
**Satisfaction with air freshness** ^**e**^	3983	0.021	-0.021–0.063	0.32	T(coef>0), RH, pressure(coef<0), Q_pers_, occupancy(coef>0), aircraft(5.coef<0)

For all models R^2^<0.1, p = 0.000, except for Nr. of lower resp. symp., p = 0.48.

^a^,^b^,^c^,^d^ see Fig 1

^e^ 1 = Very satisfied, 2 = Somewhat satisfied, 3 = Neutral, 4 = Somewhat dissatisfied, 5 = Very dissatisfied^f^ NS—not selected by the stepwise variable selection

^g^ The flight-related variables that were included in the final model based on a significance level of p<0.2 for removal from the model (stepwise backwards variable selection [41]). In parentheses the directions of the coefficients for the variables (continuous) or their categories (categorical) are indicated when significant at p<0.05.

Analyses on symptom indicators calculated for each flight further support the relationship between peak ozone concentrations and passenger-reported symptoms (S5 Table). Significant positive association was obtained for itchy eyes, feeling lightheaded or dizzy and for the maximum number of selected irritation symptoms. Consistent with the results of the previous analyses, *average* ozone concentrations demonstrated a weaker relationship with symptoms (S6 Table).

Our results agree reasonably well with the relationship between ambient ozone concentrations and building-related symptoms in the US EPA BASE study, which used a similar methodological approach [17]. The authors found a significant positive association between ambient ozone concentrations and dry eyes, headache and upper respiratory and neurological building related symptoms, with a linear concentration-response relationship with increasing ozone levels for most of these symptoms. The trend was significant for upper respiratory symptoms. The mean outdoor ozone concentration for the 100 buildings included in the BASE study was ~33 ppb (range 2–98 ppb). Although indoor ozone concentrations were not measured for the BASE buildings, given the measured ventilation rates one would expect the indoor ozone levels to be 10–50% of the outdoor levels [53,54]. In other words, the indoor ozone concentrations in the BASE buildings and in the aircraft monitored in the present study were likely comparable, although high-end values were probably larger in the aircraft cabins than in the BASE buildings.

The first studies indicating potential health effects of ozone in aircraft date back to the 1960’s and 1970’s [20, 37, 55–57]. Reed at al. [35] reported three to four times more frequent ozone-related symptoms in aircraft flying at high altitudes than in aircraft at low altitudes. The frequency of such symptoms was shown to be significantly associated with flights on Boeing 747SP aircraft, which were designed for higher-altitude flight than conventional 747 aircraft and thus had potentially greater ozone exposure [36]. De Ree et al. [58] examined ozone concentrations in airliner cabins on 24 polar routes evenly distributed between two airlines. The aircraft for one of the airlines had no catalytic converters and were fitted with humidifiers, whereas the aircraft for the other airline had catalytic converters but no humidifiers. The percentage of crewmembers who reported ozone-related symptoms did not differ significantly between the two airlines and ozone levels did not correlate significantly with changes in symptoms. However, a major limitation was that smoking was permitted on the flights that used ozone converters but not on flights without converters. Additionally, the ozone and relative humidity data were incomplete in these studies. More recently, 29 subjects were exposed to two levels of ozone (<2 and 60–80 ppb) at two outside air supply rates (2.4 and 4.7 l/s per person) in flights simulated in a section of a B-767 aircraft cabin reconstructed inside a climate chamber [26]. The subjects judged the air quality and 12 of the symptoms (including eye and nasal irritation, lip and skin dryness, headache, dizziness, mental tension, claustrophobia) to be significantly worse for the “ozone” condition compared to the “no ozone” condition.

Our study population may be too small to confidently support some of the outcomes. The large number of tests may have resulted in a few associations appearing by chance. Another limitation of this study is the assignment of the measured environmental parameters to all passengers sharing the environment on the respective flight, while the analyses used the individual passenger as the unit of analysis. However, related studies have used this method with confidence and did not find a meaningful effect of adjustment for potential correlations between subjects sharing a space [17, 59]. Moreover, the consistency in the indicated relationships across the various methodological approaches we used, including tests on aggregate variables constructed for each flight, and the similarity of our findings to previous studies provide a high degree of confidence in the results.

### Covariates in the models

The flight related variables most consistently significant in the regression models where ozone was significant, were temperature, relative humidity, flight duration, occupancy load and airline. Medium and long flight duration was significantly associated with more irritation symptoms as well as muscular and digestive symptoms, compared to short flights. All symptoms may be exacerbated by longer flight durations, most notably in going from short to medium distances [52, 60]. Reed et al. [35] reported that symptoms associated with ozone were significantly associated with flight duration and type of aircraft. In our study, passengers on wide-body aircraft (presumably flying at higher altitude) tended to have higher odds of having dry eyes and eye pain than on standard body aircraft (B737). However, having any ear and head related symptom, rating of the air quality and several symptom categories aggregated per flight exhibited a negative odds ratio/coefficient for wide-body aircraft (Tables 4 and 5, S5 and S6 Tables).

Although low relative humidity in aircraft cabins has been suggested to be a factor for mucosal irritation, especially ocular, nasal, and dermal symptoms and headache [31, 34, 60, 61], in our study no significant association was shown between low relative humidity and dry, irritated throat or dry mouth and lips. The inability of subjects to perceive low humidity as symptoms of dryness has been widely discussed in the literature [34, 62]. In a recent study, Grün et al. [62] concluded that relative humidity does not explain the dryness complaints in a simulated aircraft cabin. However, relative humidity in our study was positively associated with several other symptoms. This is consistent with the observation of a strong inverse correlation between ventilation rate and relative humidity (ρ = -0.62), which indicates that higher relative humidity in our study was associated with less outside air supply and thus, higher concentration of indoor pollutants. Our findings match those from a carefully controlled chamber study by Strøm-Tejsen et al. [63] which found that increasing the relative humidity by reducing the outdoor ventilation rate increased the complaints of headache, dizziness, and claustrophobia. This was most likely due to the increased level of contaminants at the reduced ventilation rate.

Ventilation rate was significant for several of the symptom variables. Although the relationship was mostly inverse, there was no clear consistency in the direction of its effect. Studies have indicated associations between CO_2_ concentration or ventilation rate and building related symptoms [64, 65]. However, this relationship and its comparison between aircraft environment and buildings can be complicated. While increased ventilation removes indoor contaminants that may cause symptoms and limits the time available for ozone-initiated reactions to occur in the gas-phase, it may significantly increase the transport of ozone into the cabin.

Several other variables may be important when attempting to explain the observed symptoms. These include stress, work control and satisfaction, seat comfort, baseline health (prior to the flight) and history of atopy [32, 52, 66, 67].

### UFP and ozone-initiated reactions

Concentrations of ultrafine particles in the aircraft cabins were much lower than in studies that have been conducted in buildings [68, 69]. The measurements were conducted at cruising altitude where the concentration of particles outside the aircraft is low; the ventilation air in aircraft passes through high efficiency filters; and a high percentage of the air is recirculated increasing the efficacy of the filters [70]. Meal service is the major source of particles. Thirty-eight out of 58 flights with UFP data did not provide meal service. The correlation coefficient between PNC and ozone for these flights was positive, relatively strong and statistically significant, while it was negative, weak and not significant for flights with meal service (S7 Table, all data). It is instructive to examine the correlation between PNC and ozone for each of the PNC bins on flights without meal service. For the bin with PNC above 5000 cm^-3^ (two such episodes), both ozone and PNC were elevated during most of the flight. Ozone concentrations were significantly higher than those measured during low PNC periods on other flights, and PNC levels occasionally exceeded those measured in typical urban air [68]. For periods with PNC between 5000 and 500 cm^-3^, the correlation between ozone and PNC was weak. These periods lasted on average 13 minutes (12% of the total measurement time). Similar results were obtained for periods of minor and major UFP events (S8 Table). For periods with PNC under 500 cm^-3^, the correlation with ozone was relatively strong (r = 0.30), but not significant. These observations suggest an overall link between ozone and PNC across the flights, but ozone may not fully explain the UFP events. Episodic variation in UFP might be associated with changes in the Environmental Control System or variations in engine power. It was not possible to discern such events during the course of air monitoring in the cabins. The relatively low ultrafine particle levels at cruising altitude are not anticipated to adversely impact the passengers. The relationship between ozone and PNC on flights without meal service, however, demonstrates the occurrence of reactions between ozone and indoor pollutants, including human emissions, which can lead to increased concentrations of submicron particles throughout the flight [25, 71–76].

Ozone-initiated reactions can occur at ozone concentrations much lower than the current FAA regulation [11, 77, 78]. Products of these reactions have been shown to impact perceived indoor air quality [12, 13] and may have negative health effects [11, 14–16]. The high air exchange rates on commercial flights limit the time available for reactions between ozone and various gas-phase unsaturated organic compounds. We therefore anticipate that most of the ozone-initiated chemistry within aircraft cabins is surface chemistry—ozone reacting with constituents of skin oil on exposed skin, hair and clothing as well as with various unsaturated organic compounds on surfaces within the aircraft. Indeed, in simulated aircraft environments, surface reactions of ozone with humans and materials including clothing have been demonstrated to increase the levels of saturated and unsaturated aldehydes, formaldehyde, formic acid and squalene oxidation products including acetone, 6-methyl-5-hepten-2-one (6-MHO) and 4-oxopentanal (4-OPA) [79–81]. These results have been recently confirmed in measurements on actual US transcontinental and transoceanic flights [46]. Some of the oxidation products can be irritants at elevated concentrations (e.g., 4-OPA) [82].

## Conclusions

Exposure to ozone during flight may lead to discomfort and associated symptoms among passengers. The symptoms that were significantly associated with ozone concentrations were related to the eyes and the upper respiratory system. Ozone may further be related to dry mouth and poorer perceived indoor air quality. Byproducts of ozone-driven chemistry may be partly responsible for some of these symptoms. Symptoms reported by passengers may be shared by flight attendants. In light of these concerns the FAA’s 1985 regulation on cabin ozone concentrations should be revisited and action should be taken to ensure low ozone levels during entire flights. Since the performance of catalytic converters can significantly decrease during their lifetime, their mandatory use should be coupled with regular performance checks and maintenance. These recommendations are reinforced by the fact that the ozone levels measured in this study were relatively low. Future studies on ozone-related symptoms should be extended to flights where passengers are routinely exposed to higher ozone levels.

## Supporting Information

S1 Table Spearman correlation coefficients between the measured parameters and some flight characteristics (n = 83; for occupancy n = 81). (DOCX)

S2 Table Mean and maximum number of symptoms within symptom categories and satisfaction with the indoor environment. (DOCX)

S3 Table Descriptive statistics for average prevalence (%), average number of symptoms and average IAQ sensation on each flight (n = 80). (DOCX)

S4 Table Significant associations between ozone concentration (A. scaled by a factor of 10 (aORs per 10ppb), B. Ln-transformed ozone concentration) and reported symptoms/perceptions. (DOCX)

S5 Table Results of the stepwise backward linear regression analyses on the associations between Ln-transformed maximum ozone concentration and reported prevalence, average number of symptoms and IAQ sensation on each flight. (DOCX)

S6 Table Results of the stepwise backward linear regression analyses on the associations between Ln-transformed average ozone concentration and reported prevalence, average number of symptoms and IAQ sensation on each flight. (DOCX)

S7 Table Ultrafine particle concentrations and corresponding ozone levels (average for each flight) stratified over the cutoff values of 10-minute running average PNC of 500 and 5000 cm^-3^ on flights with and without meal service. (DOCX)

S8 Table Ultrafine particle concentrations and corresponding ozone levels (average for each flight) during major and minor events on flights with and without meal service. (DOCX)
