# Supplementary material for: Impact of Cabin Ozone Concentrations on Passenger Reported Symptoms in Commercial Aircraft
Source: PLoS One. 2015 May 26;10(5):e0128454. doi: 10.1371/journal.pone.0128454 (PMC4444275; doi:10.1371/journal.pone.0128454)
Supplement: S1 Table — (DOCX) [file pone.0128454.s001.docx]

*Supporting Information*

**Impact of cabin ozone concentrations on passenger reported symptoms in commercial aircraft**

**S1 Table**. **Spearman correlation coefficients between the measured parameters and some flight characteristics (n=83; for occupancy n=81).**

|  | **Mean T** | **Mean RH** | **Mean pressure** | **Mean CO_2_** | **Ln(O_3_-aver)** | **Ln(O_3_-max)** | **Ln(Q_pers_)** | **Capacity** | **Occupancy** |
| --- | --- | --- | --- | --- | --- | --- | --- | --- | --- |
| Mean T | 1 |  |  |  |  |  |  |  |  |
| Mean RH | -0.25^a^ | 1 |  |  |  |  |  |  |  |
| Mean pressure | -0.22^a^ | 0.30^a^ | 1 |  |  |  |  |  |  |
| Mean CO_2_ | 0.12 | 0.60^a^ | 0.08 | 1 |  |  |  |  |  |
| Ln(O_3_-aver) | 0.14 | -0.25^a^ | -0.07 | -0.15 | 1 |  |  |  |  |
| Ln(O_3_-max) | 0.20 | -0.36^a^ | -0.07 | -0.15 | 0.86^a^ | 1 |  |  |  |
| Ln(Q_pers_) | -0.11 | -0.62^a^ | -0.09 | -0.98^a^ | 0.12 | 0.14 | 1 |  |  |
| Capacity^b^ | -0.35^a^ | 0.15 | 0.43^a^ | -0.09 | -0.15 | -0.06 | 0.09 | 1 |  |
| Occupancy | 0.12 | 0.62^a^ | 0.28^a^ | 0.68^a^ | -0.09 | -0.13 | -0.69^a^ | 0.08 | 1 |

^a^ p<0.05; ^b^ number of seats
