# Supplementary material for: Impact of Cabin Ozone Concentrations on Passenger Reported Symptoms in Commercial Aircraft
Source: PLoS One. 2015 May 26;10(5):e0128454. doi: 10.1371/journal.pone.0128454 (PMC4444275; doi:10.1371/journal.pone.0128454)
Supplement: S2 Table — (DOCX) [file pone.0128454.s002.docx]

**Impact of cabin ozone concentrations on passenger reported symptoms in commercial aircraft**

**S2 Table. Mean and maximum number of symptoms within symptom categories and satisfaction with the indoor environment.**

| **Symptom group** | **N** | **Mean** | **Max** |
| --- | --- | --- | --- |
| Nr. of all symp.^a^ | 4158 | 2.37 | 20 |
| Nr. of irritation symp.^b^ | 4158 | 0.89 | 8 |
| Nr. of eye&upper resp. symp.^c^ | 4158 | 1.11 | 9 |
| Nr. of muscular symp. | 4158 | 0.64 | 8 |
| Nr. of ear, head symp.^c^ | 4158 | 0.39 | 6 |
| Nr. of digestive symp. | 4158 | 0.11 | 4 |
| Nr. of neurol. symp. | 4158 | 0.09 | 4 |
| Nr. of lower resp. symp. | 4158 | 0.02 | 2 |
| Rating of air quality ^d^ | 4068 | 2.38 | 5 |
| Satisfaction with odor ^e^ | 4079 | 2.02 | 5 |
| Satisfaction with air freshness ^e^ | 4097 | 2.18 | 5 |

^a^ number of all symptoms in the questionnaire (see full list under various symptom groups in the Methods)
^b^ number of the following symptoms: watery eyes, itchy eyes, dry eyes, blurred dim altered vision, eye pain, runny nose or sneezing, dry irritated or sore throat, hoarseness/loss of voice, cough
^c^ nose bleed and sinus pain/pressure/congestion were included among the ear, head symptoms in these tests, not among eye and upper respiratory symptoms
^d^ 1=Very good, 2=Good, 3=Adequate, 4=Poor, 5=Very poor
^e^ 1=Very satisfied, 2=Somewhat satisfied, 3=Neutral, 4=Somewhat dissatisfied, 5=Very dissatisfied
