# Supplementary material for: Impact of Cabin Ozone Concentrations on Passenger Reported Symptoms in Commercial Aircraft
Source: PLoS One. 2015 May 26;10(5):e0128454. doi: 10.1371/journal.pone.0128454 (PMC4444275; doi:10.1371/journal.pone.0128454)
Supplement: S3 Table — (DOCX) [file pone.0128454.s003.docx]

**Impact of cabin ozone concentrations on passenger reported symptoms in commercial aircraft**

**S3 Table. Descriptive statistics for average prevalence (%), average number of symptoms and average IAQ sensation on each flight (n=80).**

| **Symptom** | **Mean** | **Min.** | **Max.** |
| --- | --- | --- | --- |
| Dry mouth/lips | 25.3 | 0 | 45.3 |
| Dry eyes | 21.1 | 0 | 53.8 |
| Itchy eyes | 9.1 | 0 | 30.8 |
| Dry, irritated, sore throat | 7.4 | 0 | 27.3 |
| Headache | 6.5 | 0 | 26.7 |
| Runny nose, sneezing | 5.8 | 0 | 22.4 |
| Cough | 4.5 | 0 | 22.2 |
| Watery eyes | 4.3 | 0 | 12.5 |
| Blurred, dim, altered, vision | 1.7 | 0 | 9.1 |
| Lightheaded/dizzy/faint | 1.3 | 0 | 7.9 |
| Eye pain | 1.2 | 0 | 15.4 |
| Hoarseness | 0.9 | 0 | 6.7 |
| Heart pounding | 0.3 | 0 | 5.9 |
| Any eye mouth symp. | 45.8 | 0 | 78.3 |
| Any muscular symptom | 34.9 | 0 | 61.8 |
| Any upper resp. symptom | 33.3 | 0 | 64.5 |
| Any ear, head symptom | 24.8 | 0 | 58.8 |
| Any digestive symptom | 9.7 | 0 | 30.4 |
| Any neurol. symptom | 7.4 | 0 | 27.6 |
| Any lower resp. symptom | 1.5 | 0 | 12.5 |
| Av.nr.of all symp ^a^ | 2.35 | 0 | 4.83 |
| Av.nr.of irritation symp.^b^ | 0.89 | 0 | 1.63 |
| Max.nr.of irritation symp.^b^ | 3.9 | 0 | 8.0 |
| Av.nr. of eye&upper resp. symp.^c^ | 1.10 | 0 | 2.26 |
| Av.nr. of muscular symp. | 0.64 | 0 | 1.46 |
| Av.nr. of ear, head symp.^c^ | 0.39 | 0 | 0.90 |
| Av.nr. of digestive symp. | 0.11 | 0 | 0.38 |
| Av.nr. of neurol. symp. | 0.09 | 0 | 0.34 |
| Av.nr. of lower resp. symp. | 0.02 | 0 | 0.13 |
| Rating of air quality ^d^ | 2.37 | 2.0 | 2.9 |
| Satisfaction with odor ^e^ | 2.03 | 1.4 | 2.6 |
| Satisfaction with air freshness ^e^ | 2.17 | 1.5 | 2.9 |

^a^ number of all symptoms in the questionnaire (see full list under various symptom groups in the Methods)
^b^ number of the following symptoms: watery eyes, itchy eyes, dry eyes, blurred dim altered vision, eye pain, runny nose or sneezing, dry irritated or sore throat, hoarseness/loss of voice, cough
^c^ nose bleed and sinus pain/pressure/congestion were included among the ear, head symptoms in these tests, not among eye and upper respiratory symptoms
^d^ 1=Very good, 2=Good, 3=Adequate, 4=Poor, 5=Very poor
^e^ 1=Very satisfied, 2=Somewhat satisfied, 3=Neutral, 4=Somewhat dissatisfied, 5=Very dissatisfied
