# Supplementary material for: Impact of Cabin Ozone Concentrations on Passenger Reported Symptoms in Commercial Aircraft
Source: PLoS One. 2015 May 26;10(5):e0128454. doi: 10.1371/journal.pone.0128454 (PMC4444275; doi:10.1371/journal.pone.0128454)
Supplement: S4 Table — (DOCX) [file pone.0128454.s004.docx]

**Impact of cabin ozone concentrations on passenger reported symptoms in commercial aircraft**

**S4 Table. Significant associations between ozone concentration (A. scaled by a factor of 10 (aORs per 10ppb), B. Ln-transformed ozone concentration) and reported symptoms/perceptions.** Results from linear regression analyses are shown in italics. All regression models were adjusted for T, RH, cabin pressure, Q_pers_, percent occupancy, airline, duration, aircraft type, sex, smoking habits and age (full models). The flight related variables that were significant in the final models (p<0.05) are listed in the last column. In parentheses the directions of the ORs (dichotomous outcome variable) and coefficients (continuous outcome variable, *italic*) for the variables (continuous) or their significant categories (categorical) are indicated.

**A.**

| **Dependent Variable** | **N** | **aOR/**  ***coeff.*** | **95% CI** | **p-value** | **Significant flight related variables** |
| --- | --- | --- | --- | --- | --- |
| Maximum ozone concentration | | | | | |
| Dry mouth or lips | 3940 | 1.02 | 1.00 – 1.05 | 0.030 | occupancy (OR<1), airline (3.OR>1) |
| Any eye, mouth symptom | 3940 | 1.02 | 1.00 – 1.05 | 0.018 | RH(OR>1), Q_pers_(OR<1), occupancy(OR<1), airline(2,3.OR>1), duration(2,3.OR>1), aircraft(2,3,4.OR>) |
| *Nr. of irritation symp.^a^* | *3940* | *0.013* | *0.002 – 0.023* | *0.017* | *RH(coef>0), occupancy(coef<0), airline(2,3.coef>0), duration(2,3.coef>0), aircraft(2,3,4.coef>0)* |
| *Nr. of eye&upper resp. symp.*^b^ | *3940* | *0.018* | *0.005 – 0.030* | *0.006* | *RH(coef>0), occupancy(coef<0), airline(2,3.coef>0), duration(2,3.coef>0), aircraft(2,4.coef>0)* |
| Average ozone concentration | | | | | |
| Itchy eyes | 3940 | 1.09 | 1.02 – 1.16 | 0.007 | duration(2.OR>1) |
| Eye pain | 3867 | 1.26 | 1.06 – 1.49 | 0.008 | T(OR>1), aircraft(4,5.OR>1) |
| Any eye, mouth symptom | 3940 | 1.07 | 1.02 – 1.12 | 0.003 | RH(OR>1), Q_pers_(OR<1), occupancy(OR<1), airline(2,3.OR>1), duration(2,3.OR>1), aircraft (2,3,4,5.OR>1) |
| *Nr. of irritation symp.^a^* | *3940* | *0.027* | *0.0028 – 0.051* | *0.029* | *RH(coef>0), occupancy (coef<0), airline(2,3.coef>0), duration(2,3.coef>0), aircraft(2,3,4.coef>0)* |
| *Nr. of eye&upper resp. symp.* ^b^ | *3940* | *0.043* | *0.014 – 0.072* | *0.003* | *T(coef<0), occupancy(coef<0), airline(2,3.coef>0), duration(2,3.coef>0), aircraft(2,4.coef>0)* |
| *Rating of air quality* ^c^ | *3878* | *0.022* | *0.005 – 0.039* | *0.009* | *T(coef>0), occupancy(coef>0),* |

**B.**

| **Dependent Variable** | **N** | **aOR/**  ***coeff.*** | **95% CI** | **p-value** | **Other flight related variables significant in the model (p<0.05)** |
| --- | --- | --- | --- | --- | --- |
| Maximum ozone concentration | | | | | |
| *Nr. of eye&upper resp. symp.*^b^ | *3940* | *0.067* | *0.008 – 0.126* | *0.026* | *T(coef<0), RH(coef>0), occupancy(coef<0), airline(2,3.coef>0), duration(2,3.coef>0), aircraft(3.coef>0)* |
| *Rating of air quality* ^c^ | *3878* | *0.041* | *0.007 – 0.075* | *0.018* | *T(coef>0), occupancy(coef>0),* |
| Average ozone concentration | | | | | |
| Itchy eyes | 3940 | 1.17 | 1.02 – 1.35 | 0.027 | duration(2.OR>1) |
| Any lower resp. symptom | 3867 | 0.71 | 0.54 – 0.95 | 0.020 | - |
| *Nr. of lower resp. symp.* | *3940* | *-0.008* | *-0.014 – -0.002* | *0.008* | *RH(coef>0)* |
| *Rating of air quality* ^c^ | *3878* | *0.041* | *0.011 – 0.070* | *0.007* | *T(coef>0), occupancy(coef>0),* |

For all models R^2^<0.1, except Eye pain, R^2^=0.11

For all models p=0.000, except Itchy eyes (scaled average O_3_), p=0.09; Itchy eyes (Ln average O_3_), p=0.12; Any lower resp. symp. (Ln average O_3_), p=0.83; Nr. of lower resp. symp. (Ln average O_3_), p=0.72
^a^ number of the following symptoms: watery eyes, itchy eyes, dry eyes, blurred dim altered vision, eye pain, runny nose or sneezing, dry irritated or sore throat, hoarseness/loss of voice, cough; Continuous – third column presents coefficients (*italic*)
^b^ nose bleed and sinus pain/pressure/congestion were included among the ear, head symptoms in these tests, not among eye and upper respiratory symptoms; Continuous – third column presents coefficients (*italic*)

^c^ 1=Very good, 2=Good, 3=Adequate, 4=Poor, 5=Very poor; Continuous – third column presents coefficients (*italic*)
