# Supplementary material for: Impact of Cabin Ozone Concentrations on Passenger Reported Symptoms in Commercial Aircraft
Source: PLoS One. 2015 May 26;10(5):e0128454. doi: 10.1371/journal.pone.0128454 (PMC4444275; doi:10.1371/journal.pone.0128454)
Supplement: S5 Table — (DOCX) [file pone.0128454.s005.docx]

**Impact of cabin ozone concentrations on passenger reported symptoms in commercial aircraft**

**S5 Table**. **Results of the stepwise backward linear regression analyses on the associations between Ln-transformed maximum ozone concentration and reported prevalence, average number of symptoms and IAQ sensation on each flight.**

| **Dependent variable** | **N** | **R2** | **Model p-value** | **MaxO_3_ (Ln)** | **Temp** | **RH** | **Press** | **Q_pers_ (Ln)** | **Occup.** | **Airline** | | **Duration** | | **Latit.^f^** | **Aircraft** | | | |
| --- | --- | --- | --- | --- | --- | --- | --- | --- | --- | --- | --- | --- | --- | --- | --- | --- | --- | --- |
|  |  |  |  |  |  |  |  |  |  | **2** | **3** | **2** | **3** | **2** | **2** | **3** | **4** | **5** |
| Dry mouth/lips | 80 | 0.08 | **0.048** |  |  |  |  |  |  | 0.0023 | 0.068 |  |  |  |  |  |  |  |
| Dry eyes | 78 | 0.23 | 0.053 | 0.012 | 0.015 | 0.006 |  |  | **-0.16** |  |  | **0.063** | 0.046 |  | 0.076 | 0.03 | 0.076 | 0.083 |
| Itchy eyes | 80 | 0.13 | **0.01** | **0.014** |  |  |  |  |  |  |  | **0.037** | 0.022 |  |  |  |  |  |
| Dry, irritated, sore throat | 78 | 0.21 | 0.052 | -0.011 |  |  |  | 0.052 | 0.070 | -0.052 | -0.011 |  |  |  | -0.028 | -0.043 | -0.001 | -0.026 |
| Headache | 80 | 0.04 | 0.08 |  |  |  | 0.004 |  |  |  |  |  |  |  |  |  |  |  |
| Runny nose, sneezing | 80 | None |  |  |  |  |  |  |  |  |  |  |  |  |  |  |  |  |
| Cough | 80 | 0.15 | **0.006** | **-0.011** |  |  |  |  |  | -0.017 | **-0.028** |  |  |  |  |  |  |  |
| Watery eyes | 80 | 0.15 | 0.28 |  | -0.003 | 0.002 |  |  |  | 0.035 | 0.030 | 0.009 | 0.015 |  | 0.022 | -0.003 | 0.012 | 0.007 |
| Blurred, dim, altered vision | 78 | 0.22 | **0.001** |  | -0.003 | 0.001 |  | **0.026** | **0.031** |  |  |  |  |  |  |  |  |  |
| Lightheaded/dizzy/faint | 78 | 0.20 | **0.012** | **0.0056** |  | **0.0024** | -0.002 |  | -0.020 |  |  | 0.0036 | 0.0112 |  |  |  |  |  |
| Eye pain | 80 | 0.26 | **0.001** |  | **0.005** | -0.001 |  |  |  |  |  |  |  |  | 0.005 | 0.009 | **0.024** | 0.018 |
| Hoarseness | 78 | 0.072 | 0.06 | 0.0030 |  |  |  |  | 0.012 |  |  |  |  |  |  |  |  |  |
| Heart pounding | 80 | 0.09 | 0.21 |  |  |  |  |  |  |  |  |  |  | 0.0042 | -0.003 | -0.003 | **-0.007** | -0.006 |
| Any eye mouth symp. | 80 | 0.08 | 0.10 |  |  |  | 0.016 |  |  |  |  | 0.082 | 0.067 |  |  |  |  |  |
| Any muscular symptom | 80 | 0.074 | 0.45 |  |  |  |  |  |  |  |  | 0.064 | 0.053 |  | 0.027 | -0.016 | -0.090 | -0.035 |
| Any upper resp. symptom | 80 | None |  |  |  |  |  |  |  |  |  |  |  |  |  |  |  |  |
| Any ear, head symptom | 80 | 0.10 | 0.14 |  | -0.016 |  |  |  |  |  |  |  |  |  | -0.041 | -0.029 | -0.083 | **-0.151** |
| Any digestive symptom | 78 | 0.18 | **0.012** |  | **-0.009** |  | 0.006 |  | -0.049 |  |  | 0.013 | 0.032 |  |  |  |  |  |
| Any neurol. symptom | 80 | 0.09 | **0.026** |  |  |  | **0.0066** | 0.025 |  |  |  |  |  |  |  |  |  |  |
| Any lower resp. symptom | 80 | 0.054 | 0.52 | -0.005 |  |  |  |  |  |  |  |  |  |  | -0.005 | -0.006 | -0.005 | -0.017 |
| Av.nr.of all symp ^a^ | 80 | 0.05 | 0.13 |  |  |  |  |  |  | 0.304 | **0.665** |  |  |  |  |  |  |  |
| Av.nr.of irritation symp.^b^ | 80 | 0.06 | 0.099 |  |  |  |  |  |  |  |  | 0.158 | 0.235 |  |  |  |  |  |
| Max.nr.of irritation symp.^b^ | 80 | 0.25 | **0.001** | **0.435** |  | **0.094** |  |  |  | 0.094 | 0.95 | 0.68 | 1.16 |  |  |  |  |  |
| Av.nr. of eye&upper resp.  symp.^c^ | 80 | 0.07 | 0.21 |  |  |  |  |  |  | 0.066 | 0.276 | 0.15 | 0.12 |  |  |  |  |  |
| Av.nr. of muscular symp. | 80 | 0.14 | 0.13 |  |  |  |  |  |  |  |  | 0.136 | 0.188 | 0.131 | 0.003 | -0.091 | **-0.308** | -0.208 |
| Av.nr. of ear, head symp.^c^ | 80 | 0.09 | 0.23 |  | -0.020 |  |  |  |  |  |  |  |  |  | -0.037 | -0.058 | -0.152 | **-0.230** |
| Av.nr. of digestive symp. | 80 | 0.22 | **0.001** |  | -0.008 |  | **0.008** | **0.059** |  |  |  |  |  | **0.039** |  |  |  |  |
| Av.nr. of neurol. symp. | 80 | 0.10 | 0.08 |  |  |  | 0.004 | 0.033 |  | 0.012 | 0.039 |  |  |  |  |  |  |  |
| Av.nr. of lower resp. symp. | 80 | 0.13 | 0.06 | **-0.011** |  |  |  |  |  |  |  |  |  |  | -0.004 | -0.021 | -0.005 | **-0.043** |
| Rating of air quality ^d^ | 78 | 0.29 | **0.000** |  |  | 0.006 | -0.013 |  | 0.137 |  |  |  |  | **-0.193** |  |  |  |  |
| Satisfaction with odor ^e^ | 80 | 0.24 | **0.02** | 0.027 | **0.032** | 0.0074 |  |  |  | -0.331 | -0.171 |  |  |  | -0.068 | -0.099 | -0.099 | -0.206 |
| Satisfaction with air freshness ^e^ | 80 | 0.15 | **0.007** |  | **0.039** | **0.011** |  |  |  |  |  |  |  | **-0.113** |  |  |  |  |

Coefficients for the flight related variables included in the model based on a significance level of p<0.2 for removal from the model, are listed. Significance at p<0.05 is indicated in bold.
^a^ number of all symptoms in the questionnaire (see full list under various symptom groups below)
^b^ number of the following symptoms: watery eyes, itchy eyes, dry eyes, blurred dim altered vision, eye pain, runny nose or sneezing, dry irritated or sore throat, hoarseness/loss of voice, cough
^c^ nose bleed and sinus pain/pressure/congestion were included among the ear, head symptoms in these tests, not among eye and upper respiratory symptoms
^d^ 1=Very good, 2=Good, 3=Adequate, 4=Poor, 5=Very poor
^e^ 1=Very satisfied, 2=Somewhat satisfied, 3=Neutral, 4=Somewhat dissatisfied, 5=Very dissatisfied
^f^ The effect of latitude was tested only when the stepwise variable selection excluded ozone concentration from the model
