# Supplementary material for: Impact of Cabin Ozone Concentrations on Passenger Reported Symptoms in Commercial Aircraft
Source: PLoS One. 2015 May 26;10(5):e0128454. doi: 10.1371/journal.pone.0128454 (PMC4444275; doi:10.1371/journal.pone.0128454)
Supplement: S6 Table — (DOCX) [file pone.0128454.s006.docx]

**Impact of cabin ozone concentrations on passenger reported symptoms in commercial aircraft**

**S6 Table**. **Results of the stepwise backward linear regression analyses on the associations between Ln-transformed average ozone concentration and reported prevalence, average number of symptoms and IAQ sensation on each flight.**

| **Dependent variable** | **N** | **R2** | **Model p-value** | **Ave O_3_ (Ln)** | **Temp** | **RH** | **Press** | **Q_pers_ (Ln)** | **Occup.** | **Airline** | | **Duration** | | **Latit.** | **Aircraft** | | | |
| --- | --- | --- | --- | --- | --- | --- | --- | --- | --- | --- | --- | --- | --- | --- | --- | --- | --- | --- |
|  |  |  |  |  |  |  |  |  |  | **2** | **3** | **2** | **3** | **2** | **2** | **3** | **4** | **5** |
| Dry eyes | 78 | 0.16 | **0.027** |  | 0.011 | 0.066 |  |  | **-0.163** |  |  | **0.084** | **0.107** |  |  |  |  |  |
| Itchy eyes | 80 | 0.15 | **0.007** | **0.012** |  |  |  |  |  |  |  | **0.043** | 0.027 |  |  |  |  |  |
| Dry, irritated, sore throat | 78 | 0.24 | **0.006** | **-0.014** |  |  |  | 0.050 | 0.071 |  |  |  |  |  | 0.009 | -0.003 | **0.041** | -0.002 |
| Cough | 80 | 0.21 | **0.001** | **-0.011** |  |  |  |  |  | -0.009 | -0.020 |  |  |  |  |  |  |  |
| Lightheaded/dizzy/faint | 78 | 0.15 | 0.067 | 0.0023 |  | **0.0021** | -0.001 |  | -0.018 |  |  | 0.0041 | 0.0098 |  |  |  |  |  |
| Hoarseness | 78 | 0.06 | 0.10 | 0.0019 |  |  |  |  | 0.0112 |  |  |  |  |  |  |  |  |  |
| Any lower resp. symptom | 80 | 0.11 | 0.19 | **-0.005** |  |  | 0.0021 |  |  |  |  |  |  |  | -0.015 | -0.011 | -0.012 | -0.023 |
| Max.nr.of irritation symp. | 80 | 0.21 | **0.006** | 0.222 |  | **0.076** |  |  |  | -0.025 | 0.846 | 0.72 | 1.13 |  |  |  |  |  |
| Av.nr. of lower resp. symp. | 80 | 0.14 | **0.043** | **-0.009** |  |  |  |  |  |  |  |  |  |  | -0.009 | -0.020 | -0.011 | **-0.045** |
| Satisfaction with odor | 80 | 0.28 | **0.004** |  | 0.029 | 0.0088 |  |  |  | -0.334 | -0.133 |  |  | **-0.129** | -0.060 | -0.094 | -0.029 | -0.24 |

Coefficients for the flight related variables included in the model based on a significance level of p<0.2 for removal from the model, are listed. Significance at p<0.05 is indicated in bold. The table includes the final models that are different from those in Table S5.
