# Supplementary material for: Impact of Cabin Ozone Concentrations on Passenger Reported Symptoms in Commercial Aircraft
Source: PLoS One. 2015 May 26;10(5):e0128454. doi: 10.1371/journal.pone.0128454 (PMC4444275; doi:10.1371/journal.pone.0128454)
Supplement: S7 Table — (DOCX) [file pone.0128454.s007.docx]

**Impact of cabin ozone concentrations on passenger reported symptoms in commercial aircraft**

**S7 Table. Ultrafine particle concentrations and corresponding ozone levels (average for each flight) stratified over the cutoff values of 10-minute running average PNC of 500 and 5000 cm^-3^ on flights with and without meal service.**

|  | **All flights** | | **No meal service** | | **Meal service** | |
| --- | --- | --- | --- | --- | --- | --- |
|  | **PNC(cm^-3^)** | **O_3_(ppb)** | **PNC(cm^-3^)** | **O_3_(ppb)** | **PNC(cm^-3^)** | **O_3_(ppb)** |
| *All data* | | | | | | |
| Nr. of flights | 58 | | 38 | | 20 | |
| Aver. measurement duration(min) | 196 | 216 | 108 | 131 | 361 | 379 |
| Median | 64 | 10 | 50 | 13 | 134 | 9 |
| Max. | 22052 | 110 | 22052 | 110 | 5984 | 57 |
| Correl. coef. | **0.37** | | **0.45** | | -0.22 | |
| *Periods with 10-min running mean above 5000 cm^-3^* | | | | | | |
| Nr. of flights | 7 | | 2 | | 5 | |
| Aver. duration (min) | 38 | | 68 | | 25 | |
| Aver. fraction of tot. meas. time | 0.29 | | 0.81 | | 0.09 | |
| Median | 19108 | 5 | 27979 | 74 | 17358 | 5 |
| Max. | 32804 | 97 | 32804 | 97 | 24082 | 6 |
| Correl. coef. | 0.74 | | 1.0 | | -0.33 | |
| *Periods with 10-min running mean between 500 and 5000 cm^-3^* | | | | | | |
| Nr. of flights | 24 | | 12 | | 12 | |
| Aver. duration (min) | 24 | | 13 | | 15 | |
| Aver. fraction of tot. meas. time | 0.10 | | 0.12 | | 0.08 | |
| Median | 1061 | 6 | 942 | 18 | 1361 | 5 |
| Max. | 3105 | 115 | 3105 | 115 | 2702 | 52 |
| Correl. coef. | -0.07 | | 0.09 | | -0.24 | |
| *Periods with 10-min running mean below 500 cm^-3^* | | | | | | |
| Nr. of flights | 58 | | 38 | | 20 | |
| Aver. duration (min) | 192 | | 112 | | 345 | |
| Aver. fraction of tot. meas. time | 0.95 | | 0.95 | | 0.95 | |
| Median | 54 | 10 | 46 | 12 | 83 | 10 |
| Max. | 250 | 118 | 250 | 118 | 157 | 50 |
| Correl. coef. | 0.12 | | 0.30 | | -0.26 | |

Significant Pearson’s correlation coefficients are in bold.
