# Supplementary material for: Impact of Cabin Ozone Concentrations on Passenger Reported Symptoms in Commercial Aircraft
Source: PLoS One. 2015 May 26;10(5):e0128454. doi: 10.1371/journal.pone.0128454 (PMC4444275; doi:10.1371/journal.pone.0128454)
Supplement: S8 Table — (DOCX) [file pone.0128454.s008.docx]

**Impact of cabin ozone concentrations on passenger reported symptoms in commercial aircraft**

**S8 Table. Ultrafine particle concentrations and corresponding ozone levels (average for each flight) during major and minor events on flights with and without meal service.**

|  | **All flights** | | **No meal service** | | **Meal service** | |
| --- | --- | --- | --- | --- | --- | --- |
|  | **PNC(cm^-3^)** | **O_3_(ppb)** | **PNC(cm^-3^)** | **O_3_(ppb)** | **PNC(cm^-3^)** | **O_3_(ppb)** |
| *Major events* | | | | | | |
| Nr. of events | 17 | | 3 | | 14 | |
| Aver. duration (min) | 28 | | 52 | | 23 | |
| Median | 4279 | 6 | 23345 | 52 | 4117 | 5 |
| Max. | 40485 | 95 | 28749 | 95 | 40485 | 59 |
| Correl. coef. | 0.33 | | 0.84 | | -0.17 | |
| *Minor events* | | | | | | |
| Nr. of events | 46 | | 17 | | 29 | |
| Aver. duration (min) | 16 | | 17 | | 15 | |
| Median | 399 | 6 | 376 | 6 | 563 | 8 |
| Max. | 1312 | 128 | 1153 | 128 | 1312 | 85 |
| Correl. coef. | -0.08 | | 0.08 | | -0.18 | |
